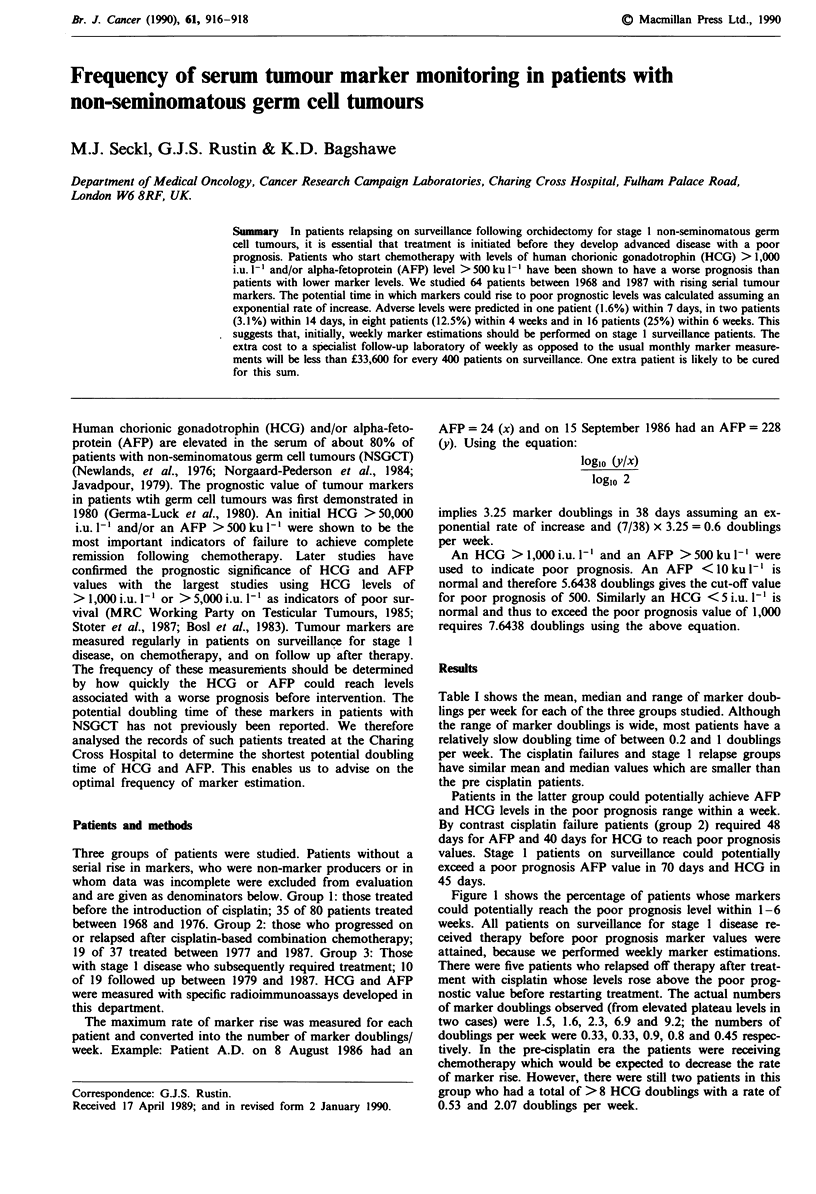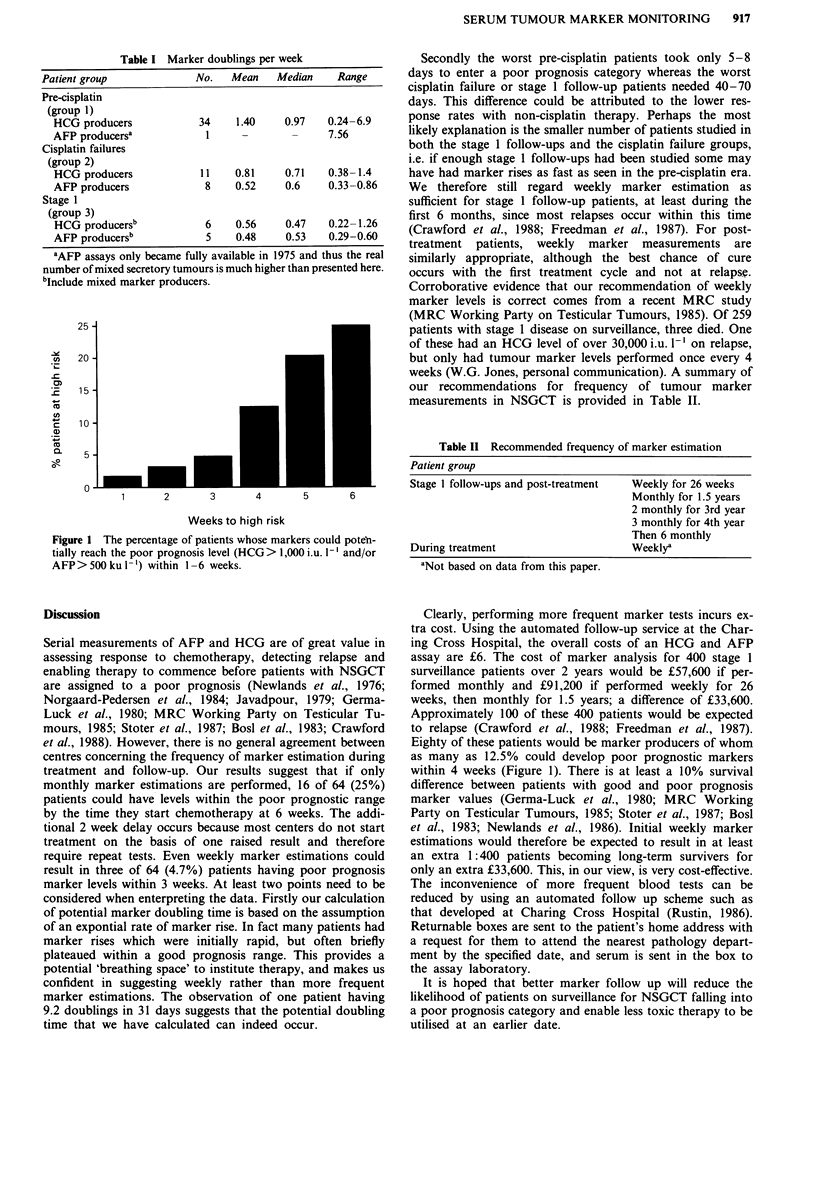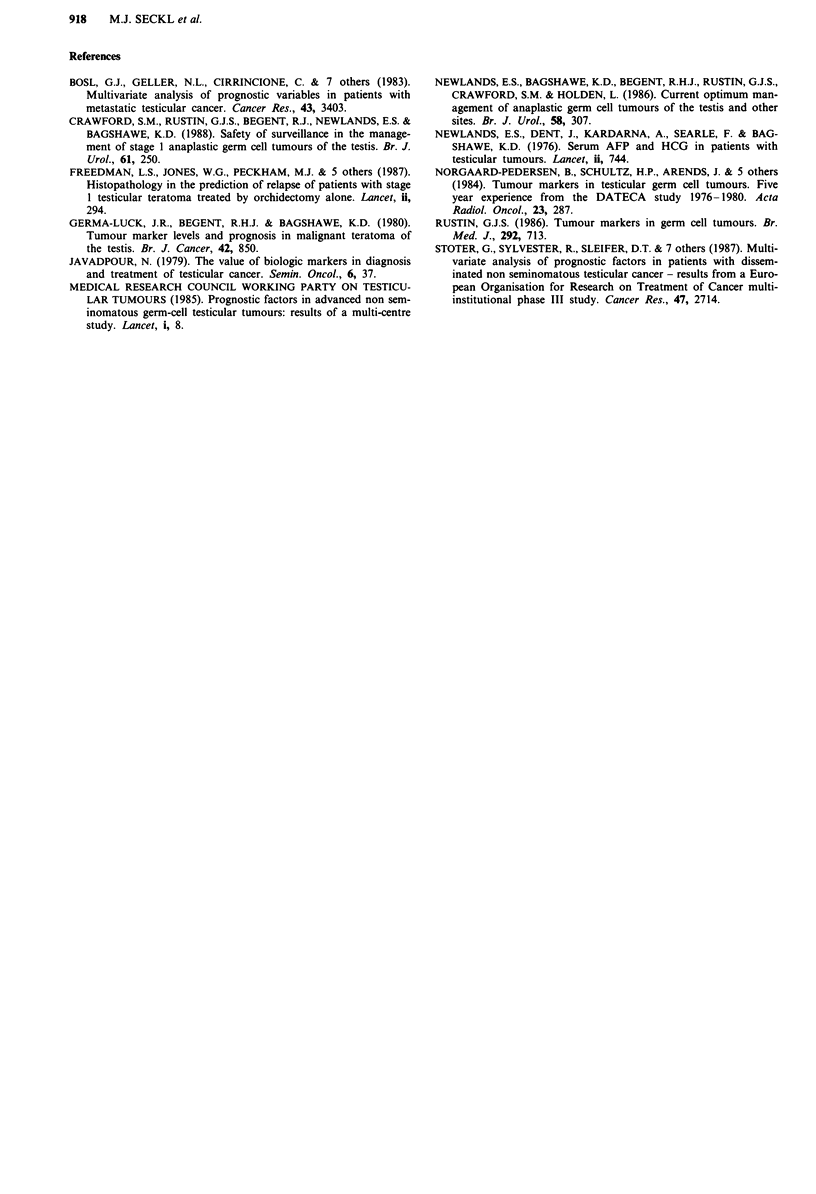# Frequency of serum tumour marker monitoring in patients with non-seminomatous germ cell tumours.

**DOI:** 10.1038/bjc.1990.205

**Published:** 1990-06

**Authors:** M. J. Seckl, G. J. Rustin, K. D. Bagshawe

**Affiliations:** Department of Medical Oncology, Cancer Research Campaign Laboratories, Charing Cross Hospital, London, UK.

## Abstract

In patients relapsing on surveillance following orchidectomy for stage 1 non-seminomatous germ cell tumours, it is essential that treatment is initiated before they develop advanced disease with a poor prognosis. Patients who start chemotherapy with levels of human chorionic gonadotrophin (HCG) greater than 1,000 i.u. l-1 and/or alpha-fetoprotein (AFP) level greater than 500 ku l-1 have been shown to have a worse prognosis than patients with lower marker levels. We studied 64 patients between 1968 and 1987 with rising serial tumour markers. The potential time in which markers could rise to poor prognostic levels was calculated assuming an exponential rate of increase. Adverse levels were predicted in one patient (1.6%) within 7 days, in two patients (3.1%) within 14 days, in eight patients (12.5%) within 4 weeks and in 16 patients (25%) within 6 weeks. This suggests that, initially, weekly marker estimations should be performed on stage 1 surveillance patients. The extra cost to a specialist follow-up laboratory of weekly as opposed to the usual monthly marker measurements will be less than 33,600 pounds for every 400 patients on surveillance. One extra patient is likely to be cured for this sum.


Br. J. Cancer (1990), 61, 916–918 © Macmillan Press Ltd., 1990

### Frequency of serum tumour marker monitoring in patients with non-seminomatous germ cell tumours

M.J. Seckl, G.J.S. Rustin & K.D. Bagshawe

*Department of Medical Oncology, Cancer Research Campaign Laboratories, Charing Cross Hospital, Fulham Palace Road, London W6 8RF, UK.*

**Summary** In patients relapsing on surveillance following orchidectomy for stage 1 non-seminomatous germ cell tumours, it is essential that treatment is initiated before they develop advanced disease with a poor prognosis. Patients who start chemotherapy with levels of human chorionic gonadotrophin (HCG) $>1,000$ i.u. $l^{-1}$ and/or alpha-fetoprotein (AFP) level $>500$ ku $l^{-1}$ have been shown to have a worse prognosis than patients with lower marker levels. We studied 64 patients between 1968 and 1987 with rising serial tumour markers. The potential time in which markers could rise to poor prognostic levels was calculated assuming an exponential rate of increase. Adverse levels were predicted in one patient (1.6%) within 7 days, in two patients (3.1%) within 14 days, in eight patients (12.5%) within 4 weeks and in 16 patients (25%) within 6 weeks. This suggests that, initially, weekly marker estimations should be performed on stage 1 surveillance patients. The extra cost to a specialist follow-up laboratory of weekly as opposed to the usual monthly marker measurements will be less than £33,600 for every 400 patients on surveillance. One extra patient is likely to be cured for this sum.

Human chorionic gonadotrophin (HCG) and/or alpha-fetoprotein (AFP) are elevated in the serum of about 80% of patients with non-seminomatous germ cell tumours (NSGCT) (Newlands, *et al.*, 1976; Norgaard-Pederson *et al.*, 1984; Javadpour, 1979). The prognostic value of tumour markers in patients wtih germ cell tumours was first demonstrated in 1980 (Germa-Luck *et al.*, 1980). An initial HCG $>50,000$ i.u. $l^{-1}$ and/or an AFP $>500$ ku $l^{-1}$ were shown to be the most important indicators of failure to achieve complete remission following chemotherapy. Later studies have confirmed the prognostic significance of HCG and AFP values with the largest studies using HCG levels of $>1,000$ i.u. $l^{-1}$ or $>5,000$ i.u. $l^{-1}$ as indicators of poor survival (MRC Working Party on Testicular Tumours, 1985; Stoter *et al.*, 1987; Bosl *et al.*, 1983). Tumour markers are measured regularly in patients on surveillance for stage 1 disease, on chemotherapy, and on follow up after therapy. The frequency of these measurements should be determined by how quickly the HCG or AFP could reach levels associated with a worse prognosis before intervention. The potential doubling time of these markers in patients with NSGCT has not previously been reported. We therefore analysed the records of such patients treated at the Charing Cross Hospital to determine the shortest potential doubling time of HCG and AFP. This enables us to advise on the optimal frequency of marker estimation.

### Patients and methods

Three groups of patients were studied. Patients without a serial rise in markers, who were non-marker producers or in whom data was incomplete were excluded from evaluation and are given as denominators below. Group 1: those treated before the introduction of cisplatin; 35 of 80 patients treated between 1968 and 1976. Group 2: those who progressed on or relapsed after cisplatin-based combination chemotherapy; 19 of 37 treated between 1977 and 1987. Group 3: Those with stage 1 disease who subsequently required treatment; 10 of 19 followed up between 1979 and 1987. HCG and AFP were measured with specific radioimmunoassays developed in this department.

The maximum rate of marker rise was measured for each patient and converted into the number of marker doublings/week. Example: Patient A.D. on 8 August 1986 had an AFP = 24 ($x$) and on 15 September 1986 had an AFP = 228 ($y$). Using the equation:

$$\frac{\log_{10}(y/x)}{\log_{10} 2}$$

implies 3.25 marker doublings in 38 days assuming an exponential rate of increase and $(7/38) \times 3.25 = 0.6$ doublings per week.

An HCG $>1,000$ i.u. $l^{-1}$ and an AFP $>500$ ku $l^{-1}$ were used to indicate poor prognosis. An AFP $<10$ ku $l^{-1}$ is normal and therefore 5.6438 doublings gives the cut-off value for poor prognosis of 500. Similarly an HCG $<5$ i.u. $l^{-1}$ is normal and thus to exceed the poor prognosis value of 1,000 requires 7.6438 doublings using the above equation.

### Results

Table I shows the mean, median and range of marker doublings per week for each of the three groups studied. Although the range of marker doublings is wide, most patients have a relatively slow doubling time of between 0.2 and 1 doublings per week. The cisplatin failures and stage 1 relapse groups have similar mean and median values which are smaller than the pre cisplatin patients.

Patients in the latter group could potentially achieve AFP and HCG levels in the poor prognosis range within a week. By contrast cisplatin failure patients (group 2) required 48 days for AFP and 40 days for HCG to reach poor prognosis values. Stage 1 patients on surveillance could potentially exceed a poor prognosis AFP value in 70 days and HCG in 45 days.

Figure 1 shows the percentage of patients whose markers could potentially reach the poor prognosis level within 1–6 weeks. All patients on surveillance for stage 1 disease received therapy before poor prognosis marker values were attained, because we performed weekly marker estimations. There were five patients who relapsed off therapy after treatment with cisplatin whose levels rose above the poor prognostic value before restarting treatment. The actual numbers of marker doublings observed (from elevated plateau levels in two cases) were 1.5, 1.6, 2.3, 6.9 and 9.2; the numbers of doublings per week were 0.33, 0.33, 0.9, 0.8 and 0.45 respectively. In the pre-cisplatin era the patients were receiving chemotherapy which would be expected to decrease the rate of marker rise. However, there were still two patients in this group who had a total of $>8$ HCG doublings with a rate of 0.53 and 2.07 doublings per week.

Correspondence: G.J.S. Rustin.
Received 17 April 1989; and in revised form 2 January 1990.

**Table I** Marker doublings per week

| *Patient group* | *No.* | *Mean* | *Median* | *Range* |
|---|---|---|---|---|
| Pre-cisplatin (group 1) | | | | |
| HCG producers | 34 | 1.40 | 0.97 | 0.24–6.9 |
| AFP producers[a] | 1 | – | – | 7.56 |
| Cisplatin failures (group 2) | | | | |
| HCG producers | 11 | 0.81 | 0.71 | 0.38–1.4 |
| AFP producers | 8 | 0.52 | 0.6 | 0.33–0.86 |
| Stage 1 (group 3) | | | | |
| HCG producers[b] | 6 | 0.56 | 0.47 | 0.22–1.26 |
| AFP producers[b] | 5 | 0.48 | 0.53 | 0.29–0.60 |

[a]AFP assays only became fully available in 1975 and thus the real number of mixed secretory tumours is much higher than presented here. [b]Include mixed marker producers.

**Figure 1** The percentage of patients whose markers could potentially reach the poor prognosis level (HCG > 1,000 i.u. $l^{-1}$ and/or AFP > 500 ku $l^{-1}$) within 1–6 weeks.

Secondly the worst pre-cisplatin patients took only 5–8 days to enter a poor prognosis category whereas the worst cisplatin failure or stage 1 follow-up patients needed 40–70 days. This difference could be attributed to the lower response rates with non-cisplatin therapy. Perhaps the most likely explanation is the smaller number of patients studied in both the stage 1 follow-ups and the cisplatin failure groups, i.e. if enough stage 1 follow-ups had been studied some may have had marker rises as fast as seen in the pre-cisplatin era. We therefore still regard weekly marker estimation as sufficient for stage 1 follow-up patients, at least during the first 6 months, since most relapses occur within this time (Crawford *et al.*, 1988; Freedman *et al.*, 1987). For post-treatment patients, weekly marker measurements are similarly appropriate, although the best chance of cure occurs with the first treatment cycle and not at relapse. Corroborative evidence that our recommendation of weekly marker levels is correct comes from a recent MRC study (MRC Working Party on Testicular Tumours, 1985). Of 259 patients with stage 1 disease on surveillance, three died. One of these had an HCG level of over 30,000 i.u. $l^{-1}$ on relapse, but only had tumour marker levels performed once every 4 weeks (W.G. Jones, personal communication). A summary of our recommendations for frequency of tumour marker measurements in NSGCT is provided in Table II.

**Table II** Recommended frequency of marker estimation

| *Patient group* | |
|---|---|
| Stage 1 follow-ups and post-treatment | Weekly for 26 weeks<br>Monthly for 1.5 years<br>2 monthly for 3rd year<br>3 monthly for 4th year<br>Then 6 monthly |
| During treatment | Weekly[a] |

[a]Not based on data from this paper.

### Discussion

Serial measurements of AFP and HCG are of great value in assessing response to chemotherapy, detecting relapse and enabling therapy to commence before patients with NSGCT are assigned to a poor prognosis (Newlands *et al.*, 1976; Norgaard-Pedersen *et al.*, 1984; Javadpour, 1979; Germa-Luck *et al.*, 1980; MRC Working Party on Testicular Tumours, 1985; Stoter *et al.*, 1987; Bosl *et al.*, 1983; Crawford *et al.*, 1988). However, there is no general agreement between centres concerning the frequency of marker estimation during treatment and follow-up. Our results suggest that if only monthly marker estimations are performed, 16 of 64 (25%) patients could have levels within the poor prognostic range by the time they start chemotherapy at 6 weeks. The additional 2 week delay occurs because most centers do not start treatment on the basis of one raised result and therefore require repeat tests. Even weekly marker estimations could result in three of 64 (4.7%) patients having poor prognosis marker levels within 3 weeks. At least two points need to be considered when enterpreting the data. Firstly our calculation of potential marker doubling time is based on the assumption of an expontial rate of marker rise. In fact many patients had marker rises which were initially rapid, but often briefly plateaued within a good prognosis range. This provides a potential 'breathing space' to institute therapy, and makes us confident in suggesting weekly rather than more frequent marker estimations. The observation of one patient having 9.2 doublings in 31 days suggests that the potential doubling time that we have calculated can indeed occur.

Clearly, performing more frequent marker tests incurs extra cost. Using the automated follow-up service at the Charing Cross Hospital, the overall costs of an HCG and AFP assay are £6. The cost of marker analysis for 400 stage 1 surveillance patients over 2 years would be £57,600 if performed monthly and £91,200 if performed weekly for 26 weeks, then monthly for 1.5 years; a difference of £33,600. Approximately 100 of these 400 patients would be expected to relapse (Crawford *et al.*, 1988; Freedman *et al.*, 1987). Eighty of these patients would be marker producers of whom as many as 12.5% could develop poor prognostic markers within 4 weeks (Figure 1). There is at least a 10% survival difference between patients with good and poor prognosis marker values (Germa-Luck *et al.*, 1980; MRC Working Party on Testicular Tumours, 1985; Stoter *et al.*, 1987; Bosl *et al.*, 1983; Newlands *et al.*, 1986). Initial weekly marker estimations would therefore be expected to result in at least an extra 1:400 patients becoming long-term survivers for only an extra £33,600. This, in our view, is very cost-effective. The inconvenience of more frequent blood tests can be reduced by using an automated follow up scheme such as that developed at Charing Cross Hospital (Rustin, 1986). Returnable boxes are sent to the patient's home address with a request for them to attend the nearest pathology department by the specified date, and serum is sent in the box to the assay laboratory.

It is hoped that better marker follow up will reduce the likelihood of patients on surveillance for NSGCT falling into a poor prognosis category and enable less toxic therapy to be utilised at an earlier date.

### References

BOSL, G.J., GELLER, N.L., CIRRINCIONE, C. & 7 others (1983). Multivariate analysis of prognostic variables in patients with metastatic testicular cancer. *Cancer Res.*, **43**, 3403.

CRAWFORD, S.M., RUSTIN, G.J.S., BEGENT, R.J., NEWLANDS, E.S. & BAGSHAWE, K.D. (1988). Safety of surveillance in the management of stage 1 anaplastic germ cell tumours of the testis. *Br. J. Urol.*, **61**, 250.

FREEDMAN, L.S., JONES, W.G., PECKHAM, M.J. & 5 others (1987). Histopathology in the prediction of relapse of patients with stage 1 testicular teratoma treated by orchidectomy alone. *Lancet*, **ii**, 294.

GERMA-LUCK, J.R., BEGENT, R.H.J. & BAGSHAWE, K.D. (1980). Tumour marker levels and prognosis in malignant teratoma of the testis. *Br. J. Cancer*, **42**, 850.

JAVADPOUR, N. (1979). The value of biologic markers in diagnosis and treatment of testicular cancer. *Semin. Oncol.*, **6**, 37.

MEDICAL RESEARCH COUNCIL WORKING PARTY ON TESTICULAR TUMOURS (1985). Prognostic factors in advanced non seminomatous germ-cell testicular tumours: results of a multi-centre study. *Lancet*, **i**, 8.

NEWLANDS, E.S., BAGSHAWE, K.D., BEGENT, R.H.J., RUSTIN, G.J.S., CRAWFORD, S.M. & HOLDEN, L. (1986). Current optimum management of anaplastic germ cell tumours of the testis and other sites. *Br. J. Urol.*, **58**, 307.

NEWLANDS, E.S., DENT, J., KARDARNA, A., SEARLE, F. & BAGSHAWE, K.D. (1976). Serum AFP and HCG in patients with testicular tumours. *Lancet*, **ii**, 744.

NORGAARD-PEDERSEN, B., SCHULTZ, H.P., ARENDS, J. & 5 others (1984). Tumour markers in testicular germ cell tumours. Five year experience from the DATECA study 1976–1980. *Acta Radiol. Oncol.*, **23**, 287.

RUSTIN, G.J.S. (1986). Tumour markers in germ cell tumours. *Br. Med. J.*, **292**, 713.

STOTER, G., SYLVESTER, R., SLEIFER, D.T. & 7 others (1987). Multivariate analysis of prognostic factors in patients with disseminated non seminomatous testicular cancer – results from a European Organisation for Research on Treatment of Cancer multi-institutional phase III study. *Cancer Res.*, **47**, 2714.